# The Design and Application of Target-Focused Compound Libraries

**DOI:** 10.2174/138620711795767802

**Published:** 2011-07

**Authors:** C. John Harris, Richard D Hill, David W Sheppard, Martin J Slater, Pieter F.W Stouten

**Affiliations:** 1 BioFocus Founder and Scientific Advisor, Lydith, High Street, Eynsford, Kent, DA4 0AB, UK; 2 BioFocus, Chesterford Research Park, CB10 1XL, UK; 3 Cresset Group, BioPark, AL7 3AX, UK; 4 Galapagos NV, Generaal De Wittelaan L11 A3, 2800 Mechelen, Belgium

**Keywords:** Library design, target focus, kinase, ion channel, GPCR, PPI, HTS, hit rate, BioFocus, Galapagos, SoftFocus.

## Abstract

Target-focused compound libraries are collections of compounds which are designed to interact with an individual protein target or, frequently, a family of related targets (such as kinases, voltage-gated ion channels, serine/cysteine proteases). They are used for screening against therapeutic targets in order to find hit compounds that might be further developed into drugs. The design of such libraries generally utilizes structural information about the target or family of interest. In the absence of such structural information, a chemogenomic model that incorporates sequence and mutagenesis data to predict the properties of the binding site can be employed. A third option, usually pursued when no structural data are available, utilizes knowledge of the ligands of the target from which focused libraries can be developed *via* scaffold hopping. Consequently, the methods used for the design of target-focused libraries vary according to the quantity and quality of structural or ligand data that is available for each target family. This article describes examples of each of these design approaches and illustrates them with case studies, which highlight some of the issues and successes observed when screening target-focused libraries.

## INTRODUCTION

Identifying novel and robust chemical starting points remains one of the biggest challenges in drug discovery today. Over the last decade, it has been common practice during the early stage of a project to screen vast numbers of compounds in high-throughput assays in order to identify those chemicals which have the potential to modulate the target of interest. The costly nature of such mass screening, the consequent need to use reductionist assays that are optimized primarily for scale and speed, and the increasing realization that drug property space is far from random has more recently led to the use of smaller, higher quality screening collections. The type of compounds selected for these collections is of the utmost importance.

Many strategies exist that seek to build such optimal initial screening collections. Most organizations now tend to prefer highly curated collections which have been selected to be drug-like or lead-like and to have high ligand efficiency [1]. In particular, experience has shown that eliminating compounds with undesirable molecular features (e.g. [2]), be they overt (e.g. electrophiles) or more subtle (e.g. toxicophores), reduces the incidence of false positives and improves the optimization performance downstream, thereby conserving valuable resources [3].

Several complementary strategies are employed in screening: the use of diverse small molecule libraries, target-focused libraries, natural products, and fragment collections. All of these approaches have their own particular advantages and disadvantages.

A target-focused library is a collection of compounds which has been either designed or assembled with a protein target or protein family in mind. The premise of screening such a library is that fewer compounds need to be screened in order to obtain hit compounds. Furthermore, it is generally the case that higher hit rates are observed when compared with the screening of diverse sets, and the hit clusters obtained from a successful focused library screening campaign usually exhibit discernable structure-activity relationships that facilitate follow up of these hits [4].

Focused libraries may be selected from larger, more diverse collections using computational techniques such as *in silico *docking to the target or ligand similarity calculations using molecular fingerprints. This retrospective approach to the selection of compounds has been reviewed elsewhere [5]; the focus of this article is the rational, prospective design of target-focused libraries.

Target-focused libraries are designed based upon some understanding of the target or target family of interest. The design can be based on target structural data (commonly used in the kinase, protease or nuclear receptor fields where crystallographic data are abundant), or, where target structural data are more scarce but sequence data and mutagenesis data are abundant, on broader chemogenomic principles (for example, with GPCR and ion channel targets). Alternatively, approaches based on the properties of known ligands can be deployed; these are applicable to all targets and target families provided that high quality ligand data are available – such approaches offer a useful way of “scaffold hopping” from one ligand class to another [6].

Target-focused libraries are often based around a single core or scaffold with one or more (typically 2 or 3) attachment points to which are appended specific substituents, or side chains, to arrive at the desired molecules. A scaffold diversified at two or three attachment points of diversity would, if all possible combinations were considered, generate a library of many thousands of compounds. Generally, a subset of these is chosen for synthesis, often around 100-500 compounds, selected to fully explore the design hypothesis efficiently and to adhere to drug-like properties.

When deciding the minimum size of the library several factors need to be considered including the tractability of the target, maximizing the efficiency of the chemistry and the importance of observing initial structure-activity relationships (SAR) in the hit clusters. Some guidelines related to the SAR issue have been proposed [4]. In 1999, the BioFocus group pioneered the design and commercial production of novel target-focused libraries and, since its SoftFocus^®^ range of libraries were first made available, they have contributed significantly to clients’ drug discovery efforts, leading to more than 100 patent filings [7] and nine published co-crystal structures, available from the Protein Data Bank (PDB) [8]: PDB codes 2R3A, 2R3G [9], 3F2A [10], 2C3I [11], 2PMN [12], 3IW8 [13], 3E7V, 3F2N and 3BQR. They have also contributed directly to the discovery of several clinical candidates [14]. This paper illustrates the design methods used to target several distinct gene families, outlining a variety of different approaches available and showing the success of these approaches in several case studies. In particular, it highlights how well-designed, target-focused libraries give higher hit rates than diverse collections, often offering potent and selective molecular starting-points that can dramatically reduce the subsequent hit-to-lead timescale.

Given the advantages of target-led approaches, where they are possible, over the erstwhile more popular diversity-led paradigm, it is no surprise that the use of focused libraries is increasing.

## METHODS FOR DESIGN

### General

For each individual chemotype within a library, the design process starts with the selection of a suitable scaffold, to which recognition elements (also called substituents or side chains) can be appended such that the assembled molecule is predicted to interact, in a generic sense, with the target family of interest. The assumption is that, for individual targets within the family, at least one of the possible combinations of scaffold and substituents will provide a good fit and therefore give a good screening hit. In selecting such scaffolds and substituents, it is crucial that careful consideration is given to synthetic accessibility, especially that the proposed chemistries are suitable for multiple parallel production and purification methods.

### Kinase Library Design

Protein kinases are one of the most important families of therapeutic targets with broad implications in cancer, inflammation and many other diseases. This family has been widely explored by a variety of screening techniques. When designing a library to inhibit a single kinase the process is relatively straightforward. The situation is more complex when the library is instead designed against the kinase superfamily (kinome), or major sub-families, as each individual kinase has its own unique requirements for ligand binding. Across the kinome the degree of similarity between individual kinases differs so the key binding points for ligands may vary, thus potentially impacting the usefulness of the library. One can identify a scaffold that can hit multiple kinases by docking it into known kinase structures. Docking a scaffold into all published kinase structures is neither feasible (because the docking and analysis process would be too lengthy), nor desirable (because the most popular targets are overrepresented). A better strategy is to evaluate the scaffold by docking it into a representative subset of kinases, where each member is carefully chosen to represent a group of targets. To generate this subset, BioFocus grouped all public domain crystal structures according to protein conformations (e.g., active/inactive, DFG in/DFG out) and ligand binding modes. Non-classical binding modes were included to ensure that the scaffolds evaluated could be used to design innovative libraries with novel vectors. From each group one structure was selected, for a total of 7 (Table **1**). Using multiple binding modes and multiple protein conformations is expected to implicitly account for the observed plasticity of the kinase binding site upon ligand binding. Minimally substituted versions of the scaffolds are docked without constraints into this subset of kinase structures. Each reasonable docked pose is assessed and scaffolds are accepted or rejected based, for example, on their predicted ability to bind multiple kinases in either the active or various inactive states. Emphasis then shifts towards side chains (substituents), selection of which should reflect the size and environment of the pockets which are targeted. For each panel member, the most appropriate side chains are predicted from the bound pose. Combining the results for every panel member generates a description of the size and nature of the side chains for the family. Under certain circumstances conflicting requirements result: for example, kinase 1 prefers small hydrophobes in pocket A, whereas kinase 2 prefers large, flexible polar groups in the same pocket. In this situation, both side chains are deliberately sampled within the library and it is this underlying softening concept that offers both coverage and potential selectivity within a library.

Whereas the initial design work focused on developing scaffolds with a correctly orientated hydrogen bonding donor-acceptor pair in order to mimic the binding of ATP within the hinge region, recent work has been focused on alternative binding modes, especially those associated with inactive conformations of the kinase. Three distinct approaches to kinase-focused library design are applied at BioFocus: hinge binding, DFG-out binding and invariant lysine binding.

#### Hinge Binding (ATP-Competitive; Type I [15])

The key feature of most scaffolds designed to interact with the hinge is a “syn” arrangement of adjacent hydrogen bond donor-acceptor groups [16]. The side chains of such compounds generally make additional interactions in pockets not utilized by ATP and therefore provide both additional affinity and a degree of selectivity for the target kinase. For example, one set of docking results from a pyrazolopyrimidine scaffold is shown in Fig. (**1**).

Using the example shown in Fig. (**1**), when selecting the side chains for this library, the predominant characteristics of the R^1^ group, circled in yellow, would be hydrophilic, since R^1^ is predicted to point towards the solvent pocket, and those of the R^2^ group, circled in blue, would be hydrophobic, since this is predicted to occupy the lipophilic site. Also included within the selection of substituents should be privileged groups [17] which are known to be important for binding to certain kinases. Finally, within each group of substituents, some side chains should be chosen simply to enhance the diversity of the final library, thus notionally increasing the chance of hitting other diverse kinases not well represented in the docking set.

#### DFG-Out Binding (Non-ATP-Competitive, Type II [15])

A more recent approach to the design of kinase libraries, with arguably higher selectivity potential [15, 18], has been to target the so-called DFG-out allosteric pocket adjacent to the ATP site. This pocket is formed when the activation loop of certain kinases undergoes a major conformational shift which disrupts the active conformation. BioFocus has developed a generalized binding model of the DFG-out pocket which enables the targeting of a range of related inactive kinase conformations. The key recognition element in DFG-out inhibitors is an “anti” arrangement of adjacent hydrogen bond donor-acceptor groups, such as those typically seen in an amide, urea or exocyclic aminoaza heterocycles.

#### Invariant Lysine Binding (Non-ATP-Competitive)

This design strategy is largely based on the novel binding mode observed in the co-crystal structure of a potent SoftFocus Kinase library (SFK33) compound bound to the kinase PIM-1 [11], wherein the compound binds to the catalytic lysine residue, making no obvious contact with the hinge region. SFK33 has proven to be a very prolific source of hits [11, 12] and this novel binding mode, which does not depend on the hinge sequence, provides a unique paradigm for novel library design.

Examples of scaffolds from each of these three design approaches together with their observed binding modes are shown in Fig. (**2**).

An appropriately designed scaffold can be successfully docked by adopting more than one of the binding modes listed above. In such cases, the design of the library and selection of the final substituents should take account of each predicted binding mode and any potential hybrid modes. Details on how these libraries compare to diverse collections in terms of screening success are reported later in this paper.

### Ion Channel Library Design

In contrast to protein kinases, where a vast amount of structural data is available, including the structures of ligands bound to both active and inactive forms of the enzymes, there is very limited structural information for ion channel targets. This is due in large part to the difficulty in crystallizing membrane bound proteins. Therefore when designing ion channel libraries, a strategy that is not wholly reliant on available structural data is necessary.

Two complementary methods for the design of ion channel libraries have been used at BioFocus. The first, Helical Domain Recognition Analysis (HDRA), is a chemogenomic technique used to target the pore region of voltage-gated ion channels (VGICs). The second technique is ligand-based and relies on the abundance of historical biological data in the ion channel field. It is applied primarily to ligand-gated ion channels (LGICs), where the binding site is generally not within the conserved pore region and therefore where little structural information is available. A comparison of the methods used to develop each design tool is shown in Fig. (**3**).

#### Voltage-Gated Ion Channels (VGICs)

The VGIC family of proteins all exhibit a tetrameric assembly of four individual transmembrane helical bundles that together comprise an ion-conduction pore and gating mechanism. The existence of a common general architecture across the VGIC family provides the basis for a general design strategy that can be customized to particular VGIC sub-types. The judicious use of the published VGIC structures, described e.g. in [19], in conjunction with sequence alignment, site-directed mutagenesis data and abundant ligand SAR allows a focused library approach analogous to that used in the kinase field.

The tetrameric helical assembly in calcium and sodium channels is covalently linked whereas in potassium channels this is not the case. These latter channels can therefore exist either as homo or hetero tetramers. Using information from published crystal structures and mutagenesis data, it is possible to construct a universal sequence alignment for the VGIC family which is very useful in exploring VGIC ligand recognition across all the diverse VGIC subtypes. Using these alignments and published mutagenesis data, the specific key amino acid residue positions that are implicated in binding small molecules within the pore region can be identified. In general, these residue positions are in close proximity, forming clusters either on the same or adjacent helices. These clusters constitute small pockets, which, by analysis of ligand SAR, can be associated with the preferred binding of small chemical moieties. By analyzing the amino acids present in each of these small pockets, the properties of the binding environment can be assessed (Table **2**).

Using this approach, the whole pore region of VGICs can be partitioned into small binding pockets. In Table **2**, the sequence numbering used is that from the KcSA structure but, using the sequence alignment for any ion channel of interest, the actual residues present at each highlighted position are known and therefore the overall binding characteristic of each channel can be classified according to the residues present.

The result of this approach is a generalized model of the pore region of VGICs, which is an approximation between the open and closed channel forms. Although relatively little is known about the precise movements within the pore during ion channel gating, recent crystallographic data suggests that movement is greatest at the intracellular ends of the helices and that the selectivity filter remains relatively unchanged. This is clearly illustrated in Fig. (**4**) where the crystal structures of the closed (pane a), partially-open (pane b) and open (pane c) forms of a VGIC are presented.

Using this method, the characterization of compound binding interactions within the pore can ensure the appropriate selection of substituents for library design. Furthermore, since this approach is not encumbered by considerations of state dependence, the resulting libraries have potential to provide both channel blockers and openers. Representative examples of scaffolds developed into ion channel libraries are shown in Fig. (**5**).

#### Ligand-Gated Ion Channels (LGICs)

In contrast to VGICs, the architecture across the ligand gated ion channels (LGICs) superfamily is not conserved and several sub-types exist, comprising trimeric, tetrameric and pentameric sub-units. This makes it difficult to devise a generic approach to pore-binding ligands. Furthermore, for known ligands, the binding domain within LGICs is located outside the pore region, which, being more variable and flexible, is very difficult to target using structure-based approaches. Therefore LGIC-focused library design using a chemogenomic approach such as HDRA is not feasible. However, a wealth of SAR information on LGIC ligands exists, which can be used as a starting point for library design using well-established pharmacophore approaches based on ligand structural comparisons. A more recent variant of this approach, which is proving to be very powerful, is to use a model of the molecular field of a ligand rather than its atom connectivity description to generate a common-field hypothesis of the bound conformations of a series of ligands. Once generated, this field pharmacophore can then be used to scan field databases to identify new scaffolds and therefore new chemical series. One such approach, used by BioFocus, employs the FieldTemplater software from Cresset BioMolecular Discovery [20]. FieldTemplater generates a model through alignment of multiple reference compounds which are conformationally sampled then aligned through matching of complementary field points. Fig. (**6**) shows a potent (10-100 nM range) TrpV1 ligand [21] and the resultant library scaffold for FFI01 (FieldFocus Ion channel library FFI01) designed to match the key field points.

It should be noted that by the very nature of the ligand-based field model, libraries designed using this approach will generally be constrained to target a much smaller family of proteins, which share closely-related ligand SAR, in contrast to the more generic approach that can be used when protein structural information is available. With this in mind, BioFocus has developed a series of libraries targeting transient receptor potential channels (TRPs) – a small sub-family of ~21 channels.

### GPCR Library Design

As with the VGICs, the transmembrane helical regions of the G-protein coupled receptors (GPCRs) exhibit spatially-conserved clusters of amino acids which constitute sub-pockets whose individual properties determine the binding of many of their ligands. This is true for GPCR Family A, for which the majority of agonists and antagonists are known from abundant mutagenesis data to bind into these transmembrane regions, thus allowing the design of focused libraries for these families [22]. In the case of GPCR Families B and C, this approach is also viable when targeting libraries of allosteric modulators which also bind within this trans-membrane region. However, such an approach would not be suitable to Family B and C orthosteric agonist library design since the natural ligand binding site is located on the extracellular loops [23]. Together with the kinase library area, the design and screening of GPCR-focused libraries has been one of the major efforts of many groups over the last decade, including the one at BioFocus. The library design principles and the Thematic Analysis tool developed at BioFocus to carry out the design (similar to the HDRA program) have been published previously [24]. More details, including synthesis and screening results, will be reviewed in a forthcoming paper [22].

### Protein-Protein Interactions Library Design

The field of protein-protein interactions (PPIs) is one which, until recently, has not been tackled using focused libraries, one of the few exceptions being the beta-turn peptidomimetic libraries targeting integrin binding reported a decade ago [25]. Recently, several reports have demonstrated that mimics of other protein paratopes such as alpha helices and beta sheets can also be successful in generating small molecule protein-protein interaction inhibitors (SMPPIIs) [26, 27]. These observations coupled with a rise in reports of drug-like SMPPIIs for a variety of target proteins, have led several groups, including BioFocus, to develop new approaches to the design of generic PPI libraries, as opposed to those aimed at a single protein target.

Of course, PPIs are ubiquitous in biological systems and, in order to devise a rational approach to targeting them, some level of focus is mandatory. Without further defining their physiological function, PPIs can be classified according to the types of biochemical processes in which they are involved; for example, substrate binding to kinases or acetylated histone binding to bromodomains [28]. However, a more generic and useful way to classify them is according to the secondary structural elements involved, for example where the principal interacting element is an alpha helix, a beta sheet, or perhaps the residues presented at the periphery of a beta-bend. Using such a classification approach, it is possible to design small molecules that mimic the key features found in these types of interacting elements, most notably hydrogen bonding patterns and the properties and trajectories of the amino acid side chains involved. These features can thus be incorporated into scaffold designs exactly as outlined for the kinases and voltage-gated ion channels and used to generate small molecule libraries for screening against a wide range of protein-protein interactions. Thus, PPI libraries are designed to mimic the key features found in secondary structural elements involved in protein-protein interactions.

For many years, the view was that to effectively mimic an alpha helical or beta sheet based interaction, one would require large, non-drug-like molecules. Indeed many large, nominally non-drug-like compounds have been reported as helix mimetics and terphenyl structures as mimics of the peptide backbone [29].

**Fig. (7):**
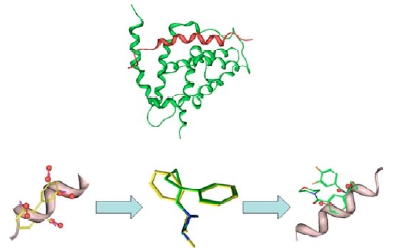
BCL-XL (green) with the peptide BAD (red); typical alpha helix structure with side chain vectors shown in red superimposed with a biphenyl (in yellow); overlay of a biphenyl with a cyclopropylphenyl, HM01 scaffold and an example of HM01 overlaid onto an alpha helix (For interpretation of the references to color in this figure legend, the reader is referred to the web version of this paper).

The approach adopted at BioFocus for the design of helix mimetic libraries is illustrated in Fig. (**7**) for a specific library scaffold (HM01). It has previously been reported that the tetra substituted biphenyl scaffold mimics the backbone of an alpha helix with the 2-,3-,2’- and 3’- side chains of the biphenyl presenting the same trajectories as the helical side chains [30]. The biphenyl scaffold itself is inherently lipophilic and non-drug-like and, moreover, offers little chance of directly gaining useful intellectual property around any hits that are obtained in screening; development of such hits would have to overcome these challenges. However, a cyclopropylphenyl scaffold would overcome some of these disadvantages, being less lipophilic (cLogP ca. 0.5 log units lower than biphenyl), and having greater potential for novelty in development. Small molecule modeling [31] has shown that a phenyl attached to a substituted cyclopropyl group, which itself contains some pi character, can mimic a biphenyl moiety with the side chains overlaying well (Fig. **7**). Such a compound could therefore be envisioned to mimic one face of an alpha helix (Generally, only one face of a helix is involved in the interaction with another protein, the opposite face usually being buried in the interior of the protein itself). Whilst the primary aim is to generate compounds with sufficient activity to interfere with a PPI selectivity is also considered. By ensuring an adequate coverage of chemical space in the selection of the substituents we anticipate that screening the library in its entirety should lead to differing selectivity patterns emerging.

Based on this series of simple observations it has been possible to devise a number of libraries that can mimic the side-chain vectors presented by alpha helices.

## CASE STUDIES

### Performance of BioFocus Kinase-Focused Libraries in Kinase Screens

Three types of kinase-focused libraries have been developed by BioFocus, namely the SoftFocus (SFK), FieldFocus (FFK) and ThemePair Fragment (TPF) libraries. These latter libraries were designed based on the scaffolds and close analogues of fully elaborated kinase-focused libraries and their average molecular weight is consequently in the 240-dalton range. The Galapagos group has screened many of these libraries against various kinases in the context of its internal research programs. The results of these screens were analyzed in terms of hit rates against 17 kinases that represent the major kinase families (AGC: 2 examples, TKL: 2, TK: 4, STE: 4, CK1: 1, CGMC: 3, and CAMK: 1). A hit is defined as a compound exhibiting more than 50% inhibition at 10 µM in a single-point, primary screening experiment and all libraries for which at least a total of 600 single-point determinations were made are included in the analysis.

The overall hit rates for all kinase-focused libraries against all 17 kinases tested is shown in Fig. (**8**). 89% of the kinase-focused libraries have higher hit rates than the diverse collection (“DIV”, 5,718 compounds), up to a hit rate of 31% for SFK52. Considering the size of the ThemePair Fragment molecules, their hit rates are surprisingly high (between 2.2 and 9.9%), indicating that kinase-focused fragments can be highly ligand efficient.

Overall hit rates are important, but consideration must also be given to the selectivity of each library against the 17 kinases. Fig. (**9**) shows a heat map for the SFK libraries. It is immediately clear that the pattern is far from homogeneous: some kinases (e.g. Kin10) are hit by many libraries, some are hit by few, but all kinases are hit by at least one library.

The individual libraries are also complementary: SFK52 hits many kinases (average hit rate 31.3%), so it is important to have SFK52 in a kinase screening collection, despite possible selectivity issues. Kin04 is hardly hit by SFK52 (1.2%), but it is hit strongly by SFK36 (22.6%), which, in terms of overall hit rate (6.8%), only takes 12th place. Had SFK36 not been included in the screening deck of Kin04, the most appealing series would have been missed. It is clear that only screening those libraries with high overall hit rates (if it is at all possible to determine that *a priori*) is not sufficient.

Of course, both the target kinase and the library may be promiscuous. Indeed, Kin10 is hit by many libraries, which may be of concern if one wants to pursue series having intrinsic selectivity versus Kin10. For Kin01, Kin03, Kin07, Kin09 and Kin17, SFK10 would serve that purpose as it does not hit Kin10. So, although SFK10 hits only 5/17 kinases with a hit rate > 3%, it is important to have it in the screening collection. The ideal composition of a set of kinase-focused libraries is actually a mixture of promiscuous and selective libraries.

To assess the hit rate distribution for a specific library, SFK49, a library with a high hit rate (16.5%), is taken as an example. It is based on the aminoimidazothiadiazole scaffold shown in Fig. (**10**).

Fig. (**11**) shows a heatmap, representing the hit rate of each of the 501 compounds of SFK49. There is a clear spread of actives over the heatmap: 138 compounds (28%) hit no kinase and 88 compounds (18%) hit only one. At the other end of the selectivity range, 4 compounds hit 75% of the kinases and 1 compound hits 80%. Clearly, this library contains compounds ranging in profile between almost completely indiscriminate and exquisitely selective. With regard to the comparative performance of the side chains of the SFK49 library, compounds with R^1^=A03 and compounds with R^2^=B07 have the highest hit rates. However, the compound with R^1^=A03 and R^2^=B07 is not the best compound in the library, indicating that R^1^ and R^2^ substitutions are not independent variables and that the combinatorial nature of the library provides important information.

In conclusion, it is clear that tailor-made, focused kinase libraries are a very valuable resource for the development of novel and selective kinase inhibitors and represent a more efficient entry to novel kinase leads than diverse screening sets.

### Performance of BioFocus Ion Channel-Focused Libraries in Ion Channel Screens

Researchers at Galapagos and BioFocus have screened a range of ion channel-focused libraries against a limited set of ion channel targets, the results of which are analyzed below.

Galapagos screened several BioFocus ion channel-focused libraries (SFI01-SFI13 and FFI01-FFI03) and a diverse vendor collection (“DIV”, 1,058 compounds) against two internal targets: a potassium VGIC (target A) and a calcium non-VGIC (target B). These assays were run in flux mode (Rb^+^ efflux for A, Ca^2+^ influx for B) at 10μM compound concentration, a hit being defined by ≥ 50% inhibition of ion flux. The primary screening data are summarized in Fig. (**12**). The compounds screened against both targets A and B are a subset (1,743 compounds) of those screened against targets A (1,858) and B (7,867).

**Fig. (12):**
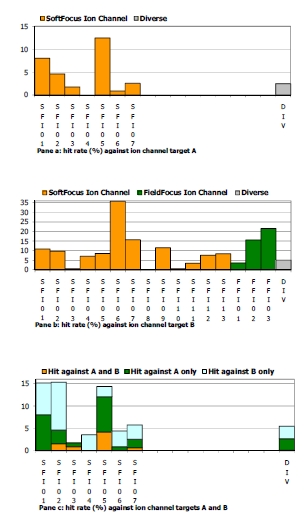
Hit rate of each ion channel-focused library against target A (pane **a**), target B (pane **b**) and targets A and B (pane **c**). For comparison, the hit rate of a diverse, vendor collection (“DIV”) is provided as well.

Of the libraries tested against both targets A and B, SFI06, SFI07, FFI02 and FFI03 in particular show much higher hit rates than the diverse set. Overall hit rates for target B are higher than for target A. For target B only 5 out of the 16 focused libraries exhibited hit rates lower than the diverse set. For target A, 4 out of the 7 focused libraries tested showed better hit rates than the vendor set, with SFI05 showing the highest hit rate. Of the compound sets tested against both targets (Fig. **12c**), SFI04 was completely selective for target B, with SFI02 and SFI06 showing weaker B selectivity. Interestingly, although the library design was not aimed at selectivity for these two channels, the large majority of the observed hit compounds were selective for one or the other channel. Conversely, at the library level, all libraries, except SFI04 and to a lesser extent SFI06, showed promiscuity for these two targets. These selectivity data mirror the more detailed observations noted above with the kinase-focused libraries.

BioFocus screened a small set of SFI01 compounds against two potassium VGICs (KCNQ2/3 and IK), one calcium VGIC (Cav) and one sodium VGIC (Nav1.5), using ion flux as a measure of activity in non voltage-clamped cells [32]. Two micromolar hits exhibited clear signs of selectivity for the calcium channel (compound A for Cav) and for one of the potassium channels (compound B for KCNQ2/3), respectively (Table **3**), demonstrating that exquisite selectivity can be obtained with a single library.

In summary, the use of specifically designed, focused ion channel libraries offers a useful alternative to the screening of diverse compound sets and appears to be an excellent means to the discovery of selective compounds across a range of channel types. As all the compounds discussed here act as inhibitors of ion currents, it remains to be seen if such libraries also offer an entry point for the development of channel activators.

## SUMMARY AND CONCLUSION

The customized design of libraries focused on the major gene families has been explored in detail, some findings from which are highlighted in this paper. Although the recycling of existing knowledge of ligands can be criticized on the grounds of intellectual property novelty, when such information is combined with new structure-based design paradigms and/or new insights into the way ligands interact with their targets, such as described herein, the observed efficiency of focused library screening becomes more understandable. Although drug property space is far from random, the use of diverse libraries will always have a place in discovery for its ability to produce the unexpected, but in terms of efficiency and, perhaps, scientific understanding, well-designed, focused libraries will continue to make a growing impact on drug discovery, as the case studies in this paper illustrate. For the future, as the understanding of structural relationships between apparently unrelated proteins increases, we expect focused libraries to be key tools for drug discovery across, as well as within, currently perceived protein target families.

## TRADEMARKS

BioFocus^®^, SoftFocus^®^, HDRA™, Helical Domain Recognition Analysis™, FieldFocus™, Thematic Analysis™, Theme Pair™, SFK™, FFK™, TPF™ and SFI™ are trademarks of Galapagos NV and/or its affiliates. FieldTemplater™ is a trademark of Cresset BioMolecular Discovery Ltd.

## Figures and Tables

**Fig. (1):**
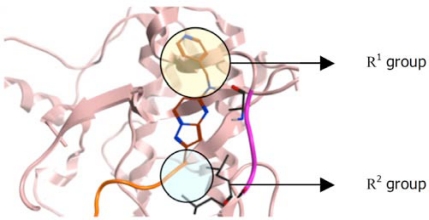
Proposed binding mode resulting from a pyrazolopyrimidine scaffold docked into Aurora A kinase. The hinge region is shown in pink and the activation loop in orange (For interpretation of the references to color in this figure legend, the reader is referred to the web version of this paper).

**Fig. (2):**
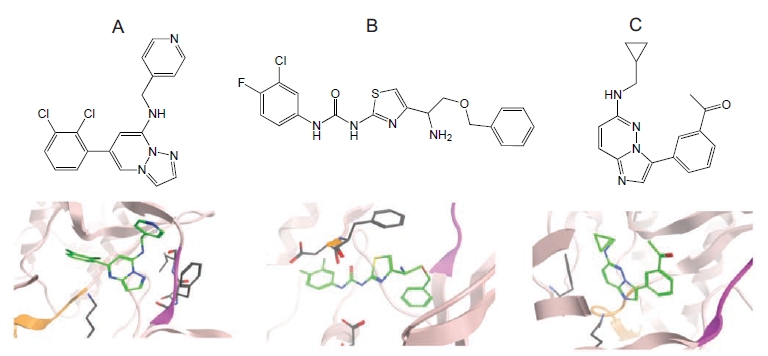
Selection of crystallized SFK library compounds in the public domain (**a**) hinge binding: SFK03 compound bound to CDK2 (PDB code 2R3F); (**b**) DFG-out binding: SFK48 compound bound to p38α [13] (PDB code 3IW8); (**c**) invariant lysine binding: SFK33 compound bound to PIM-1 [11] (PDB code 2C3I). The hinge region is shown as a purple ribbon in each case (For interpretation of the references to color in this figure legend, the reader is referred to the web version of this paper).

**Fig. (3):**
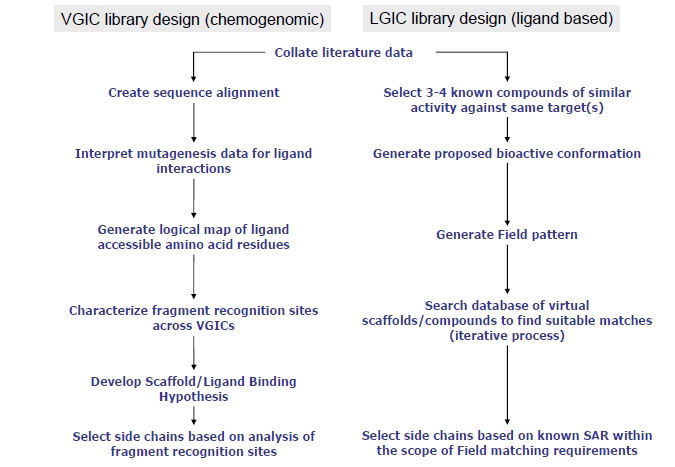
Approaches to ion channel library design.

**Fig. (4):**
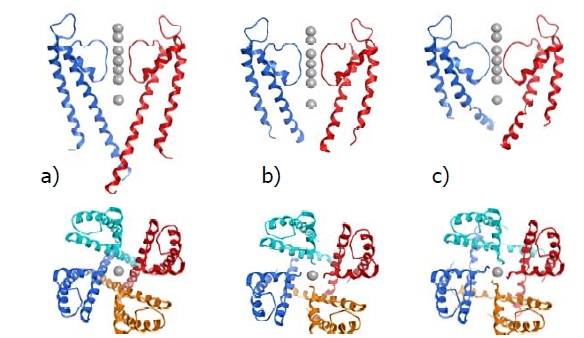
Model of voltage-dependent gating from KCSA mutant crystal structures, (**a**) closed channel state (PDB code 2JK5) viewed from the side and top respectively, (**b**) partially open state (PDB code 3F7Y) viewed from the side and top respectively and (**c**) fully open channel state (PDB code 3F5W) viewed from the side and top respectively.

**Fig. (5):**
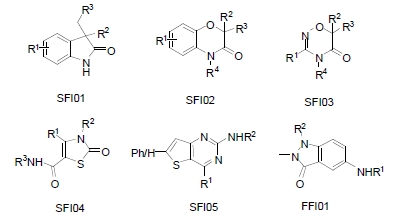
Example scaffolds. SFI (SoftFocus Ion channel) libraries are designed using HDRA to target the pore region of VGICs. FFI (FieldFocus Ion channel) libraries are designed to target LGICs using a ligand based design approach.

**Fig. (6):**
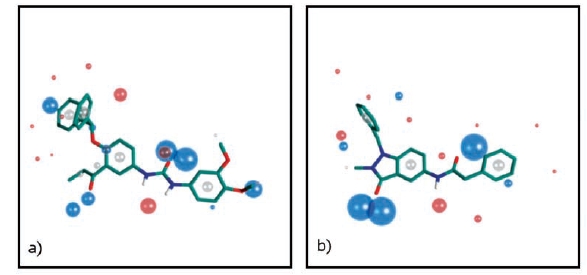
Field based scaffold library design. Template and resultant scaffold for the TRP family of ligand gated ion channels, showing field patterns for a synthetic TRPV1 agonist (**a**) and the FFI01 scaffold (**b**). Blue, red and grey spheres represent negative electrostatic, positive electrostatic and neutral hydrophobic field points, respectively (For interpretation of the references to color in this figure legend, the reader is referred to the web version of this paper).

**Fig. (8):**
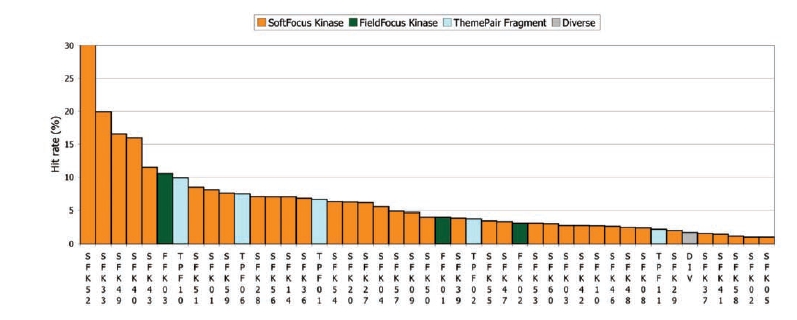
Overall hit rates for each library against all 17 kinases. For comparison, the hit rate (1.7%) of a diverse, non-specifically kinase-focused collection (“DIV”, grey bar) is provided as well. SFKs are represented by orange, FFKs by green and TPFs by cyan bars (For interpretation of the references to color in this figure legend, the reader is referred to the web version of this paper).

**Fig. (9):**
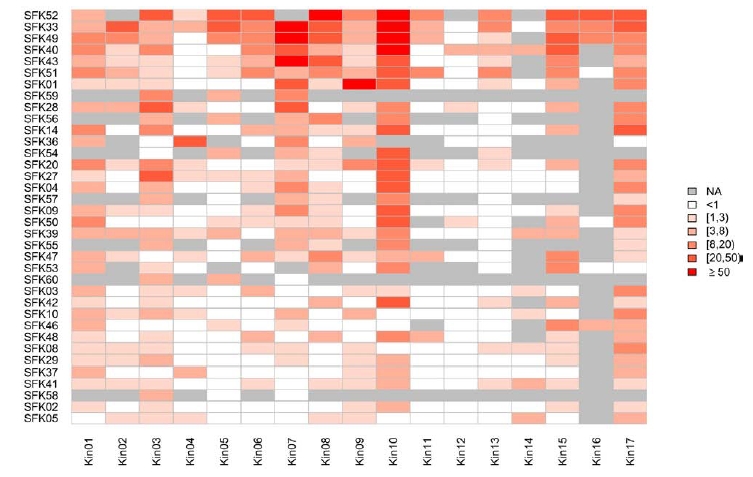
Hit rates of each library against each of the 17 kinases. Cells with hit rates > 50% are colored red. Cells with hit rates < 50% have colors ranging from white (0% hit rate) to red (50% hit rate). If fewer than 40 compounds in a library were tested against a kinase, the corresponding cell is grey (For interpretation of the references to color in this figure legend, the reader is referred to the web version of this paper).

**Fig. (10):**
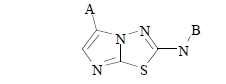
Scaffold of library SFK49 with substitution points A and B indicated.

**Fig. (11):**
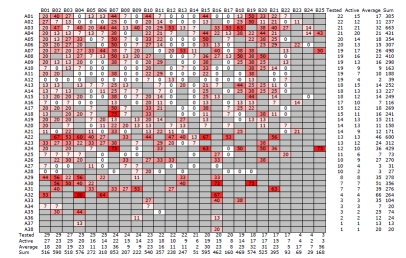
Hit rates of all compounds of library SFK49 against the 17 kinases. Substitutions in position A are on the Y-axis, in position B on the X-axis. Tested (total number of compounds tested), Active (total number of compounds that are active, i.e., that hit at least one kinase), Average (average hit rate) and Sum (sum of all hit rates) refer to all compounds in a row or column. Colors range from white (0% hit rate) to red (80% hit rate). If a compound was not synthesized or tested, the corresponding cell is grey (for interpretation of the references to color in this figure legend, the reader is referred to the web version of this paper.

**Table 1:** The Kinases with Associated Classification that Make Up the BioFocus Library Evaluation Panel

Kinase	Crystal Structure Used (PDB Access Code)	Classification
PIM-1	2C3I	Inactive conformation
MEK2	1S9I	Active conformation
P38α	1WBS	Inactive conformation
AurA	2C6E	Inactive conformation
JNK	2GMX	Active conformation
FGFR	2FGI	Active conformation
HCK	1QCF	Active conformation

**Table 2:** An Example Sub-Pocket of Five Different Channels, Showing how the Amino Acids Occupying these Defined Residue Positions Determine the Property of the Corresponding Ligand Moiety

Channel		Residue Position in Sub-Pocket (Based on KCSA Numbering)							Property of Ligand Moiety Recognized
		*75*	*99*	*100*	*100*	*103*	*104*	*107*	
Kir1.4		T	T	T	T	E	I	T	Basic amine
hERG		S	G	S	S	Y	A	F	Basic amine/aromatic ring
Cav1.1	D2 D3	G	G	N	A	L	L	F	H-bonding
Kv1.1		T	G	V	V	I	A	V	Lipophilic
Kvlqt1		T	A	V	V	F	A	A	Lipophilic

**Table 3:** IC50s (in µM) of Two SFI01 Compounds Against 4 VGICs

	KCNQ2/3	IK	Cav	Nav1.5
Compound A	>100	>100	1.74	23.64
Compound B	1.6	>100	11.66	>100
